# Whole Genome Sequence of *Treponema pallidum* ssp. *pallidum*, Strain Mexico A, Suggests Recombination between Yaws and Syphilis Strains

**DOI:** 10.1371/journal.pntd.0001832

**Published:** 2012-09-20

**Authors:** Helena Pětrošová, Marie Zobaníková, Darina Čejková, Lenka Mikalová, Petra Pospíšilová, Michal Strouhal, Lei Chen, Xiang Qin, Donna M. Muzny, George M. Weinstock, David Šmajs

**Affiliations:** 1 Department of Biology, Faculty of Medicine, Masaryk University, Brno, Czech Republic; 2 Human Genome Sequencing Center, Baylor College of Medicine, Houston, Texas, United States of America; 3 Department of Genetics, The Genome Institute, Washington University School of Medicine, St. Louis, Missouri, United States of America; Institut Pasteur, France

## Abstract

**Background:**

*Treponema pallidum* ssp. *pallidum* (TPA), the causative agent of syphilis, and *Treponema pallidum* ssp. *pertenue* (TPE), the causative agent of yaws, are closely related spirochetes causing diseases with distinct clinical manifestations. The TPA Mexico A strain was isolated in 1953 from male, with primary syphilis, living in Mexico. Attempts to cultivate TPA Mexico A strain under *in vitro* conditions have revealed lower growth potential compared to other tested TPA strains.

**Methodology/Principal Findings:**

The complete genome sequence of the TPA Mexico A strain was determined using the Illumina sequencing technique. The genome sequence assembly was verified using the whole genome fingerprinting technique and the final sequence was annotated. The genome size of the Mexico A strain was determined to be 1,140,038 bp with 1,035 predicted ORFs. The Mexico A genome sequence was compared to the whole genome sequences of three TPA (Nichols, SS14 and Chicago) and three TPE (CDC-2, Samoa D and Gauthier) strains. No large rearrangements in the Mexico A genome were found and the identified nucleotide changes occurred most frequently in genes encoding putative virulence factors. Nevertheless, the genome of the Mexico A strain, revealed two genes (TPAMA_0326 (*tp92*) and TPAMA_0488 (*mcp2-1*)) which combine TPA- and TPE- specific nucleotide sequences. Both genes were found to be under positive selection within TPA strains and also between TPA and TPE strains.

**Conclusions/Significance:**

The observed mosaic character of the TPAMA_0326 and TPAMA_0488 loci is likely a result of inter-strain recombination between TPA and TPE strains during simultaneous infection of a single host suggesting horizontal gene transfer between treponemal subspecies.

## Author Summary

*Treponema pallidum* is a Gram-negative spirochete that causes diseases with distinct clinical manifestations and uses different transmission strategies. While syphilis (caused by subspecies *pallidum*) is a worldwide venereal and congenital disease, yaws (caused by subspecies *pertenue*) is a tropical disease transmitted by direct skin contact. Currently the genetic basis and evolution of these diseases remain unknown.

In this study, we describe a high quality whole genome sequence of *T. pallidum* ssp. *pallidum* strain Mexico A, determined using the ?next generation? sequencing technique (Illumina). Although the genome of this strain contains no large rearrangements in comparison with other treponemal genomes, we found two genes which combined sequences from both subspecies *pallidum* and *pertenue*. The observed mosaic character of these two genes is likely a result of inter-strain recombination between *pallidum* and *pertenue* during simultaneous infection of a single host.

## Introduction

*Treponema pallidum* ssp. *pallidum* (TPA) and *Treponema pallidum* ssp. *pertenue* (TPE) strains, the causative agents of syphilis [1] and yaws [2], infect more than 12 and 2 million people annually, respectively [3]. Whereas syphilis is a sexually transmitted and congenital disease affecting adults and newborns worldwide, yaws is transmitted predominantly through direct skin contact and affects preferably children in warm, humid, rural areas.

During the last several years, a number of treponemal genomes have been completely sequenced including TPA Nichols (GenBank acc. no. AE000520.1 [4]), TPA SS14 (CP000805.1 [5]), TPA Chicago (CP001752.1 [6]), TPE Samoa D (CP002374.1), TPE CDC-2 (CP002375.1), TPE Gauthier (CP002376.1) [7] and *T. paraluiscuniculi* strain Cuniculi A (CP002103.1 [8]). In general, when compared to TPE strains, TPA strains differ by less than 1,200 nucleotide positions [7], [9]. Phylogenetic trees constructed from whole genome binary restriction target site data [9], from multilocus sequencing [10] and whole genome sequence alignments [10] showed a distinct clustering of TPA and TPE strains. As shown by Centurion-Lara *et al.* [11] and Gray *et al.* [12], the unusual clustering of the Mexico A TP0131 gene with several TPE strains is the result of intra-chromosomal gene conversion events. Three different alleles of the *tprD* (TP0131) gene (*D*, *D2*, and *D3*) have been identified among TPA and TPE strains [11] and the presence of individual gene alleles determines the cluster patterns [12].

The TPA Mexico A strain was isolated in 1953 from an 18-year-old male, with primary syphilis, living in Mexico [13]. Attempts to cultivate TPA Mexico A strain under *in vitro* conditions revealed a lower growth rate (compared to other tested TPA strains) and also a decreased percentage of motile treponemes compared to TPA strain Nichols [14]. The lower growth potential of Mexico A is likely to result from genetic differences between this strain and other TPA strains. Our previous study [9] revealed that the Mexico A strain contained the largest genome of all investigated TPA strains.

In this study, we compared the complete genome sequence of TPA Mexico A to complete TPA and TPE genome sequences and found a mosaic character of the Mexico A TPAMA_0326 (*tp92*) and TPAMA_0488 (*mcp2-1*) loci, i.e. having both TPA and TPE specific nucleotide sequences.

## Materials and Methods

### Preparation of chromosomal DNA

The TPA Mexico A strain used in this study was kindly provided by David L. Cox, CDC, Atlanta, GA, USA. The DNA was amplified directly from 1 µl of cells (10^5^ cells per µl) frozen in glycerol using a QIAGEN Whole Genome Amplification REPLI-g Kit (QIAGEN, Valencia, CA, USA). To separate treponemal cells from rabbit testicular cells, the samples were first centrifuged at 100×g for 5 min. Supernatant containing treponemal cells was carefully extracted and centrifuged at 14,100×g for 3 min. The resulting pellet containing treponemal cells was washed 2× in PBS buffer and centrifuged at 14,100×g for 3 min. The supernatant was removed for a final volume of 3 µl and the procedure continued according to the manufacturer's instructions. Amplified DNA was purified using a QIAEX II kit (QIAGEN, Valencia, CA, USA). The resulting DNA concentration was 602 ng/µl in a 30 µl volume.

### DNA sequencing

The chromosomal DNA was sequenced using the Illumina (Illumina, San Diego, CA, USA) technique. Several chromosomal regions of the Mexico A strain, representing sequentially related and repetitive components of treponemal genome, were amplified using a GeneAmp XL PCR kit (Applied Biosystems, Foster City, CA, USA) using the previously described TPI amplicons [9], [15]. These regions comprised the following TPI amplicons (genes): TPI-11 (*tprC*), TPI-12 (*tprD*), TPI-13B (TP0136), TPI-17A (5S, 16S and 23S rRNA [rRNA operon 1]), TPI-21B (5S, 16S and 23S rRNA [rRNA operon 2]), TPI-25A (*tprE*), TPI-25B-A (*tprF*), TPI-25B-B (*tprG*), TPI-26 (TP0326), TPI-32B (*arp*), TPI-34 (TP0470), TPI-38 (TP0488), TPI-42A (TP0548), TPI-48 (*tprI, tprJ*), TPI-66A (TP0868) and TPI-67 (*tprK*). From these XL-PCR amplicons, small insert libraries were prepared and the resulting clones were sequenced as previously described [5]. Alternatively, amplified DNA was Sanger sequenced directly using specific primers.

A set of 639 Illumina contigs (100–69,908 bp in length) and 16 Sanger contigs, resulting from sequencing of XL-PCR products, were assembled using the TPA SS14 reference genome [5]. This assembly contained 122 gaps (8.9 kb in length) in the TPA Mexico A sequence. Altogether, 117 DNA regions (containing all 122 gaps) were additionally PCR amplified and sequenced using the Sanger method.

The TP0326 (*tp92*) and TP0488 (*mcp2-1*) loci of *Treponema pallidum* subsp. *endemicum* (TEN), strain Bosnia A, were amplified using GeneAmp XL PCR kit (Applied Biosystems, Foster City, CA, USA) and Sanger sequenced using specific primers.

### Whole genome fingerprinting

The resulting genome assembly was verified using the previously described fingerprinting technique [15], [16]. The experimentally identified DNA fragments (resulting from DNA digestion at 1774 restriction target sites; [7]) were compared to the corresponding *in silico* restriction fragment lengths. The 1774 restriction target sites corresponded to a total sequence length of 10.6 kb. The average error rate of WGF was calculated previously [8] and corresponded to 27.9 bp (1.6% of the average fragment length) with a variation range between 0 and 132 bp.

### Genome annotation and G+C content calculations

Considering the close relatedness of the Mexico A and SS14 genomes (99.99% identity at the nucleotide level), the Mexico A genome was annotated according to the SS14 genome [5] with minimal gene length of 150 bp. Genes identified in the Mexico A genome were denoted with the prefix TPAMA followed by four numbers to indicate gene number. Putative virulence factors were defined as those previously described by Čejková *et al.* [7] and comprised 31 genes (including *tpr*, *arp*, and TPAMA0136 genes). All of these genes are listed in Table S1.

The G+C content was calculated in 501 bp windows using CLC Bio software (CLC Bio Katrinebjerg, Denmark).

### Nucleotide sequences accession numbers

The whole genome sequence of TPA strain Mexico A was placed in the GenBank under accession number CP003064.1. Sequences of TP0326 (*tp92*) and TP0488 (*mcp2*-1) of TEN strain Bosnia A were deposited in the GenBank under accession numbers JX392330.1 and JX392331.1, respectively.

## Results

### Whole genome sequence of the Mexico A strain and genome annotation

The genome of the Mexico A strain was determined to be 1,140,038 bp with 1,035 predicted ORFs. The final assembled genome sequence was verified using a fingerprinting technique [15], [16] where 1774 experimentally identified DNA fragments were compared to *in silico* restriction fragment lengths. No differences in fragment lengths were identified indicating correct overall assembly of the Mexico A genome. The 1774 restriction target sites corresponded to a total sequence length of 10.6 kb. Since no discrepancies between the *in silico* and the experimental restriction analysis were found (i.e. in 10.6 kb of the genome sequence out of 1,140 kb), the sequencing error rate was estimated to 10^−4^ or less.

From all annotated ORFs, 161 (15.6%) are involved in general metabolism, 125 (12.1%) in cell structure and cell processes, 51 (4.9%) in DNA replication, repair and recombination, 173 (16.7%) in regulation, transcription and translation, 113 (10.9%) in transport, and 31 (3%) in virulence. 327 ORFs (31.6%) had unknown function. In addition, 54 (5.2%) genes encoded RNAs. Coding regions represented 93.5% of the Mexico A genome. As in the SS14 (CP000805.1 [5]) and Chicago (CP001752.1 [6]) genomes, the *tprK* gene (TPAMA_0897) is represented by a number of variable sequences and the consensus sequence, therefore, contains unidentified nucleotides in these regions.

### Gene fusions and authentic frameshifts

Altogether, six genes (pseudogenes) were annotated to contain authentic frameshifts (AF) in the Mexico A genome (TPAMA_0009, TPAMA_0146, TPAMA_0316, TPAMA_0520, TPAMA_0532 and TPAMA_0812) compared to 9 genes with AF annotated in the Nichols and SS14 genomes, where 3 additional genes with AF were described (TP0217, TP0575 and TP0866). In an additional 21 cases, frameshift mutations identified in the Mexico A genome resulted in gene fusions (Table S2).

### Genomic differences between Mexico A and other syphilis treponemes

Whole genome sequence of the TPA strain Mexico A has been compared with other sequenced genomes of TPA strains including the Nichols strain (AE000520.1 [4]), SS14 (CP000805.1 [5]), and Chicago (CP001752.1 [6]) using the Lasergene software package (DNASTAR, Madison, WI, USA) and Crossmatch (P. Green, unpublished). Because of high sequence diversity, TP0131 (*tprD*) and TP0897 (*tprK*) were excluded from our calculations. The Mexico A genome differed from the SS14 genome in 175 substitutions, 85 insertions and 28 deletions, from the Chicago genome in 419 substitutions, 18 insertions and 20 deletions, and from the Nichols genome in 438 substitutions, 94 insertions and 38 deletions (ambiguously identified bases present in the Nichols genome were not counted). Changes differentiating Mexico A and Nichols genomes were found in 206 ORFs listed in Table S3. Since it is known that the Nichols and SS14 genomes contain about 200 nt errors ([10], Pospíšilová, unpublished results), we also compared the Mexico A genome with the improved version of the Nichols genome (Pospíšilová, unpublished results). From 206 ORFs originally identified as sequentially different, 138 ORFs (67%) also showed differences when compared to the improved Nichols genomic sequence. The originally identified nucleotide changes in the remaining 68 ORFs (33%) were considered to be Nichols sequencing errors. However, in the case of 14 Nichols ORFs (1.3% of the total Nichols ORFs), only partial or no sequencing data were available.

In general, the identified changes were more frequently found among genes encoding putative virulence factors and among genes involved in cell structure and processes and in genes coding for DNA replication, repair and recombination. In contrast, genes encoding components associated with general metabolism, transcription, translation, gene regulation and transport contained nucleotide changes less frequently (Table S4).

### Mosaic character of TPAMA_0326 (*tp92*) and TPAMA_0488 loci (*mcp2-1*)

**Figure 1:**
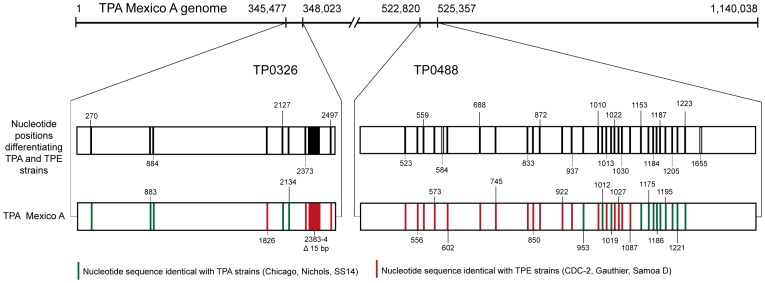
A schematic representation of the TPAMA_0326 and TPAMA_0488 loci. The Mexico A sequence was compared to three TPA (Nichols, SS14, and Chicago) and three TPE CDC-2, Gauthier, Samoa D) strains. Black vertical lines represent nucleotide positions differentiating all TPA and TPE strains. Green and red vertical lines represent nucleotide changes present in all analyzed TPA and TPE strains where the TPA Mexico A sequence is identical to TPA and TPE strains, respectively. Two nucleotide positions (584, 1655) in the TP0488 locus were found only in the Nichols and Chicago strains and not in the SS14 genome (open vertical lines). The symbol Δ stands for 15-bp deletion present in the TPE and Mexico A strains in the TP0326 gene.

In addition to TPA strains, the Mexico A genome sequence was also compared with whole genome sequences of three TPE strains including Samoa D (GenBank acc. no. CP002374.1), CDC-2 (CP002375.1) and Gauthier (CP002376.1) [7]. Of all the annotated genes, two (TPAMA_0326 (*tp92*) and TPAMA_0488 (*mcp2-1*)) showed a mosaic character, which combined sequences from both TPA and TPE strains (Fig. 1). The complete set of nucleotide changes found in the TPA and TPE regions for TP0326 and TP0488 loci are shown in Table 1 and Table 2, respectively. In the TP0326 locus, there were 8 single nucleotide positions and one 15 bp deletion that differentiated TPE strains (Samoa D, CDC-2 and Gauthier) from TPA strains (Nichols, SS14, Chicago). Out of these 9 positions, the TPAMA_0326 locus contained 5 nucleotide positions with an identical sequence to the TPA strains and 4 regions that were identical to the TPE regions, including 3 nucleotide positions and the 15 bp deletion (Fig. 1, Table 1). Similarly, the TP0488 locus contained 30 nucleotide positions that were found to be different for all analyzed TPA and TPE strains. In addition, two nucleotide positions (584, 1655) differentiated the Nichols and Chicago strains from TPE strains and from the SS14 strain. In TPAMA_0488, 12 of these 30 positions contained sequences identical to TPA strains, whereas 18 positions corresponded to sequences of TPE strains (Fig. 1, Table 2). In the remaining part of the Mexico A genome, similarities to the TPE sequences were only found in the *tprC* sequence and at two additional nucleotide positions (present in TP0314 locus and TPAMA_0319, respectively).

**Table 1:** A list of changes found in the TPAMA_0326 locus.

Mexico A coordinate (GenBank acc. no. CP003064.1)	TPAMA_0326 coordinate	Mexico A	TPE strains	TPA strains	Domain1)
345746	270 [90]	T (Y)	C (Y)	T (Y)	POTRA 1
346359	883 [295]	G (G)	A (N)	G (G)	POTRA 4
346360	884 [295]	G (G)	A (N)	G (G)	POTRA 4
347302	1826 [609]	A (Q)	A (Q)	T (L)	β-barrel domain
347603	2127 [709]	C (N)	G,T (R, E, N)2)	C (N)	β-barrel domain
347610	2134 [712]	G (G)	A (S)	G (G)	β-barrel domain
347849	2373 [791]	C (S)	C (S)	T (S)	β-barrel domain
347859–347860	2383–2384 [794–795]	deletion of 15 bp (del of 5 AA)	deletion of 15 bp (del of 5 AA)	GTAGMACCACCAGCT 3) (SSTTS, SRTTS)3)	β-barrel domain (serine residue)
347955	2479 [823]	G (A)	G (A)	C (P)	β-barrel domain

Three TPA (Nichols, SS14, and Chicago) and three TPE (CDC-2, Gauthier, and Samoa D) strains were analyzed. The numbers in square brackets correspond to amino acid position in the TPAMA_0326 protein. The amino acid sequence of the encoded protein is shown in brackets.

1) Domain prediction according to Desrosiers *et al.* [41].

2) G (R) was found in TPE CDC-2 strain, G (E) in TPE Gauthier, while T (N) was found in TPE Samoa D strain.

3) 15 bp (GTAGAACCACCAGCT) encode SRTTS in TPA Nichols and Chicago strain and 15 bp (GTAGCACCACCAGCT) encode SSTTS in TPA SS14 strain.

**Table 2:** A list of changes found at the TPAMA_0488 locus.

Mexico A coordinate (GenBank acc. no. CP003064.1)	TPAMA_0488 coordinate	Mexico A	TPE strains	TPA strains	Domain1)
523342	523 [175]	A (S)	A (S)	G (G)	-
523375	556 [186]	A (T)	A (T,I)2)	G (V)	-
523378	559 [187]	A (R)	A (R)	G (G)	-
523392	573 [191]	C (S)	C (S)	T, A (S,R)3)	-
523403	584 [195]	A (D)	A (D)	G, A (G,D)4)	-
523421	602 [201]	G (C)	G (C)	C (S)	-
523507	688 [230]	G (G)	G (G)	A (R)	-
523564	745 [249]	A (T)	A (T)	G (A)	-
523652	833 [278]	A (Q)	A (Q)	G (R)	-
523669	850 [284]	C (R)	C (R)	T (C)	-
523691	872 [291]	G (R)	G (R)	C (P)	Cache
523741	922 [308]	A (N)	A (N)	G (D)	Cache
523756	937 [313]	A (I)	A (I)	G (V)	Cache
523772	953 [318]	T (V)	C (A)	T (V)	Cache
523829	1010 [337]	A (E)	A (E)	G (G)	Cache
523831	1012 [338]	G (A)	A (T)	G (V)	Cache
523832	1013 [338]	C (A)	C (T)	T (V)	Cache
523838	1019 [340]	G (R)	A (K)	G (R)	Cache
523841	1022 [341]	G (S)	G (S)	T (I)	Cache
523846	1027 [343]	A (I)	A (I)	G (V)	Cache
523849	1030 [344]	T (F)	T (F)	A (I)	Cache
523906	1087 [363]	G (A)	G (A)	A (T)	-
523972	1153 [385]	A (R)5)	G (K)	A (E)	-
523994	1175 [392]	C (A)5)	T (T)	C (I)	-
524003	1184 [395]	A (R)	G (H)	A (R)	-
524005	1186 [396]	G (V)	A (T)	G (V)	-
524006	1187 [396]	T (V)	C (T)	T(V)	-
524014	1195 [399]	T (Y)	C (H)	T (Y)	-
524024	1205 [402]	T (L)	C (S)	T (L)	-
524040	1221 [407]	A (L)	G (S)	A (S)	-
524042	1223 [408]	C (S)	A (Y)	C (S)	-
524474	1655 [552]	A (Q)	A (Q)	G, A (R,Q)6)	-

Three TPA (Nichols, SS14, and Chicago) and three TPE (CDC-2, Gauthier, and Samoa D) strains were analyzed. Numbers in square brackets correspond to amino acid position in TPAMA_0488 protein. Amino acid sequence in the encoded protein is shown in brackets.

1) Domain prediction according to the NCBI Conserved Domain Database.

2) A (T) was identified in TPE CDC-2 strain while A (I) in TPE Gauthier and Samoa D strains.

3) T (S) was identified in TPA Nichols and Chicago strains while A (R) in TPA SS14 strain.

4) G (G) was identified in TPA Nichols and Chicago strains while A (D) in TPA SS14 strain.

5) Mexico A-specific nucleotide change in the vicinity of TPA-like change causes amino acid change specific for Mexico A.

6) G (R) was identified in TPA Nichols and Chicago strain while A (Q) in TPA SS14 strain.

### G+C content in treponemal genomes

**Figure 2:**
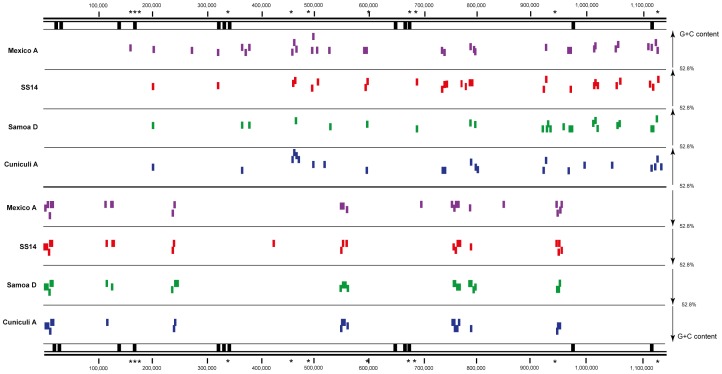
G+C content variation in selected *Treponema* strains. TPA Mexico A, TPA SS14, TPE Samoa D and *Treponema paraluiscuniculi* strain Cuniculi A were analyzed. Colored vertical lines represent 501 bp-long windows with G+C content above the 63% or below the 41% threshold. Black vertical lines represent genome locations of *tpr* genes. Stars denote 5 kb-long DNA regions with 40 or more nucleotide positions differentiating TPA and TPE strains [7]. Please note that there is no clear association of DNA regions with different G+C content and regions differentiating TPA and TPE strains [7] or locations of *tpr* genes.

The average G+C content of the Mexico A genome was found the same as for other treponemal species, 52.8%. Based on an analysis of G+C content, codon and amino acid usage, and gene positions, 77 (8.32%) of the TPA genes were predicted to be horizontally transferred [17]. To identify chromosomal regions with horizontal transfer potential, G+C content was calculated in 501 bp windows in TPA Mexico A, TPA SS14 [5], TPE Samoa D [7] and *Treponema paraluiscuniculi* strain Cuniculi A (CP002103.1 [8]) (Fig. 2). The chromosomal regions showing different G+C content (defined as G+C content higher than 63% or lower than 41%) showed a similar pattern in all four tested genomes. We compared regions with higher/lower G+C content with 5 kb-long chromosomal regions containing 40 or more nucleotide changes differentiating TPA and TPE strains which were previously identified by Čejková *et al.* [7]. From 11 such regions [7] (Fig. 2), only 3 showed significant differences in G+C content. Similarly, no clear association was found in regions with different G+C content and *tpr*-containing DNA regions.

## Discussion

Complete genome sequences of the TPA Mexico A strain was revealed. The genome size, G+C content and gene order was identical with other already sequenced TPA genomes [4]–[6]. The Mexico A genome was most closely related to SS14 genome and differed in less than 300 hundred substitutions and indels. Since it has been published that the Nichols and the SS14 genomes contain about 200 nt errors [10] a lower number of nucleotide changes differentiating the Mexico A and SS14 genome can be expected. In fact, the number of nucleotide differences between Mexico A and SS14 genomes (except of differences present in the *tprD* and *tprK* genes) is probably lower than one hundred (Pětrošová, unpublished results). In any of these comparisons, the identified differences were more frequently present in (i) genes encoding putative virulence factors, (ii) genes involved in cell structure and processes and (iii) genes coding for DNA replication, repair and recombination. In contrast, genes encoding components of general metabolism, transcription, translation, gene regulation and transport appear to be conserved.

The observed mosaic character of the Mexico A TPAMA_0326 (*tp92*) and TPAMA_0488 (*mcp2-1*) loci, combining both TPA- and TPE-specific nucleotide sequences, can be, in principle, explained by six independent mechanisms including i) an ancestral position of the Mexico A strain with respect to both TPA and TPE strains, ii) rapid accumulation of nucleotide changes during evolution of TPA strains from TPE strains with the Mexico A as an intermediate, iii) intra-strain recombination between paralogous sequences, iv) artifacts during PCR amplification (as a result of contamination with TPE genomic DNA) and/or contamination with TPE-amplified DNA, v) convergent evolution and vi) inter-strain recombination between TPA and TPE strains during simultaneous infection of one host.

**Figure 3:**
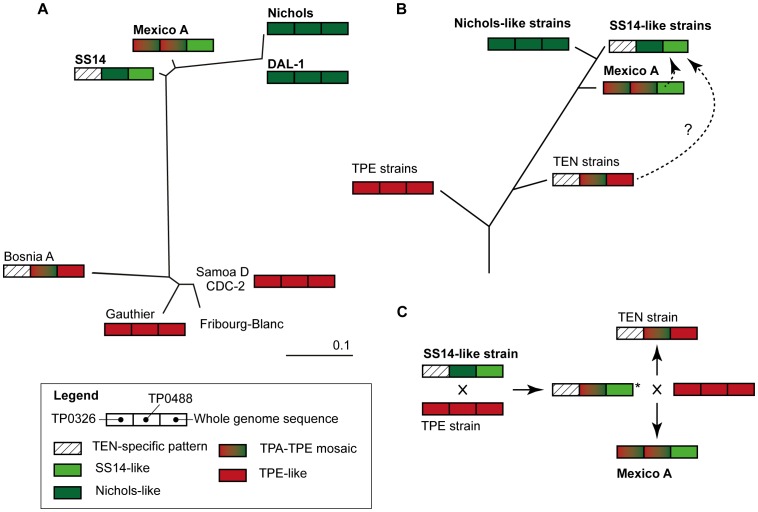
Evolutionary relationships among TP strains and hypothetical evolutionary schemes. A. Unrooted tree constructed from restriction target site data from 25.6% of the analyzed TP genomes (modified according to Šmajs *et al.* [18]). TPA (Dallas, Nichols, Mexico A and SS14), TPE (CDC-2, Gauthier and Samoa D), TEN (Bosnia A) strains and unclassified simian isolate Fribourg-Blanc were analyzed. TPA strains are shown in bold. Patterns shown for the Bosnia A strain are based on the sequences of TP0326 (GenBank acc. no. JX392330.1, this sequence is identical to partial sequence published by Harper *et al.* [19]) and TP0488 (GenBank acc. no. JX392331.1) determined by us, and on the whole genome fingerprinting data [18]. B. Scheme illustrating hypothetical evolution of TP strains. Please note that the ancestral position of Mexico A related to other TPA strains does not explain presence of TEN-specific pattern in TP0326 (*tp92*) locus in the SS14-like group of TPA strains. C. Scheme illustrating hypothetical recombinations among TP strains. A hypothetical ancestor of TEN and Mexico A strains is marked by star.

i) The first explanation can be ruled out because only two chromosomal loci (TPAMA_0326 and TPAMA_0488) showed demonstrable similarity to TPE strains. Moreover, the number of Mexico A-specific mutations (i.e., mutations that are only present in the Mexico A genome and not in other sequenced TPA genomes) is not significantly different from the number of specific mutations in other TPA genomes (data not shown). In a predicted common ancestor, one would expect a considerably higher number of ancestor-specific mutations in comparison to progenies. ii) The second hypothesis is illustrated in Fig. 3B. The hypothetical evolution scheme comprises TPA, TPE and TEN strains arranged according to their relatedness to other TP strains [18] (see also Fig. 3A). We sequenced TP0326 (*tp92*) and TP0488 (*mcp2-1*) loci in TEN strain Bosnia A (GenBank acc.no. JX392330.1 and JX392331.1, respectively; our TP0326 sequence is identical to partial *tp92* sequence of Bosnia A published by Harper *et al.* [19]). The sequencing data showed that TEN strain Bosnia A contains the same nucleotide mosaic in the TP0488 (*mcp2-1*) locus as Mexico A (with the exception of 2 single nucleotide substitutions) and similarly, some TPA isolates belonging to the SS14-like group of TPA strains show a TEN-specific pattern in the TP0326 (*tp92*) locus. It was impossible to propose an evolutionary model based only on accumulation or loss of nucleotide changes (see Fig. 3B), and this fact supports recombination hypothesis. iii) The third hypothesis was rejected when we failed to identify potential recombinant (donor) sites for the TPAMA_0326 and TPAMA_0488 genes in the Mexico A genome, despite several attempts to identify such regions using several computer programs and algorithms (RDP3, EditSeq (DNASTAR), BLAST). iv) While it is known that PCR amplification of two sequentially related templates can result in the production of chimeric DNA amplicons [20], contamination of the Mexico A genomic DNA with TPE genomic DNA can be ruled out because recombinant genes were only found for two genes of the genome. Contamination with TPE-amplified DNA (corresponding to TPAMA_0326 and TPAMA_0488 genes) was excluded based on careful analysis of Illumina reads, where no TPA- or TPE-specific Illumina reads were found in any of these regions. In fact, the presence of 15 bp-deletions in the TPAMA_0326 gene was found in all 169 individual Illumina reads covering this region. Similar analysis of the TPAMA_0488 region revealed no TPA- or TPE-specific Illumina reads; and all 37 reads, covering regions with both TPA and TPE molecular signatures, revealed the Mexico A consensus sequence. Since Illumina technology sequences individual DNA molecules, contamination of Mexico A genomic DNA with TPE PCR product can be excluded. To exclude artifacts during REPLI-g kit amplification of the Mexico A genomic DNA, three different REPLI-g amplifications were used for TPAMA_0326 and TPAMA_0488 sequencing. No discrepancies were identified during analysis of Sanger reads in these regions. Moreover, Harper *et al.* [19] sequenced partial *tp92* locus of the Mexico A strain (obtained directly from CDC, Atlanta) and the sequenced region (960 nt, GenBank acc. no. EU102088.1, containing TPE-like sequence in three nucleotide positions and a 15-bp deletion) was identical to our sequence. Sequences of TP0326 (*tp92*) from various TPE isolates published by Harper *et al.* [19] contained the 15 bp TPE-like deletion and also corresponded to TPE-like changes in the South Africa treponemal isolate. All 21 South Africa partial nucleotide sequences available in the GenBank [19] were 100% identical to the corresponding sequences of Mexico A published by Harper *et al.* [19]. Therefore, the South Africa strain appears to be another strain that is identical, or very closely related, to the Mexico A strain. Nevertheless, we found 3 nucleotide changes differentiating South Africa and Mexico A sequences published by Harper *et al.* [19] from our own sequences of Mexico A. Two of these differences were found in homopolymeric stretches (in *fliG*-tp0027 and tp0347 regions) and one SNP (C→T) was found in the *rpiA*-tp0617 region. Since both Mexico A strains came from the same laboratory (D. L. Cox, CDC Atlanta), the data suggest that possible sequencing errors in sequences published by Harper *et al.* [19] may explain these differences. To further asses the frequency of strains similar to Mexico A/South Africa, we investigated clinical samples published by Flasarová *et al.* [21] for Mexico A-specific mutations. No such nucleotide changes were found in 49 genotyped samples, indicating that the Mexico A/South Africa group of strains is not prevalent in central Europe. v) Since convergent evolution assumes acquisition of the same biological trait in unrelated lineages (operating on the level of biological function), it is extremely unlikely that it would result in exactly the same amino acid sequence of the relevant proteins. Due to degeneration of the genetic code, it is even more unlikely that convergent evolution would end up in two identical nucleotide sequences. vi) In contrast to previous alternatives, inter-strain recombination cannot be ruled out despite the fact that the probability of such event is relatively low. Moreover, the mosaic character of the TPAMA_0326 and TPAMA_0488 loci, combining both TPA- and TPE-specific nucleotide sequences, is a typical result of a recombination event after horizontal gene transfer [22]–[24]. Also, patterns found in TEN strains indicate that observed mosaics in the Mexico A genome are not artifacts, but rather the results of recombination events in the common ancestor of TPA and TEN strains (see Fig. 3C).

**Figure 4:**
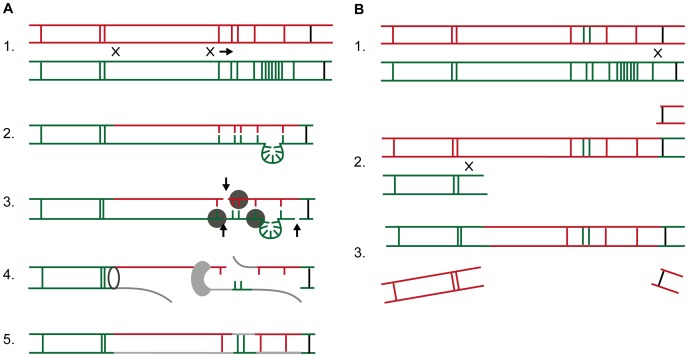
Two possible molecular mechanisms resulting in formation of the mosaic structure of the TPAMA_0326 locus. Green lines are nucleotide sequences specific for TPA strains (Nichols, SS14, Chicago), red ones for TPE strains (CDC-2, Gauthier, Samoa D). Vertical black lines denote corresponding positions of the TPAMA_0326 loci. A) Homologous recombination. 1. Homologous recombination between TPA and TPE loci with suggested possible recombination sites (x); arrow indicates direction of branch migration. 2. The resulting heteroduplex contains both TPA and TPE sequences; green loop denotes a 15 bp insertion present in all investigated TPA strains. 3. Recognition of mismatched DNA by MutS-MutL complex and subsequent cleavage of DNA strands by endonucleases (marked by arrows). 4. Unwinding of cleaved strands by DNA helicase (open elipse) and DNA polymerase-mediated (grey) gap filling. 5. Reparation of DNA breaks with DNA ligase. Alternatively, there is a possibility of an active DNA uptake across the cell membrane. Although no gene orthologs involved in natural competence have been identified in the TPA genomes, one cannot exclude this activity in one or several genes with unknown function. B) Gene conversion. Successive half crossing-over model. 1. First half crossing-over with suggested recombination site (x). 2. Successive second half crossing-over and the second recombination site (x). 3. Duplex recovery and degradation of both displaced ends. Please note that model B) requires the presence of two TPA-like nucleotide sequences in the donor TPE sequence.

There are several possible molecular mechanisms that could lead to the formation of the mosaic structure seen at the TPAMA_0326 and TPAMA_0488 loci. We propose two models (Fig. 4) that are based on the incorporation of TPE double stranded DNA. In the first model, dsDNA was integrated into the chromosome of the Mexico A ancestor through homologous recombination. The resulting DNA heteroduplex was block-repaired via mismatch repair mechanisms. Similar reparation patterns have been observed after DNA transformation of *Escherichia coli* [25] and *Helicobacter pylori* [24]. In other bacteria, mismatch repair involves the cleavage of a daughter strand by MutH, which recognizes methylated cytosine in the GATC sequence. Since TPA does not contain a MutH orthologue and no methyltransferases, the mechanism of DNA cleavage remains unknown. Both *mutS* and *mutL* have been annotated to the TPA genome.

The second mechanism is based on gene conversion events following internalization of dsDNA. Gene conversion is a common mechanism for producing antigenic variability in TPA [26]. Since TPA possesses only the RecF recombination pathway, gene conversion in TPA is likely to follow the successive half crossing-over model [27], as shown in Fig. 4. However, the mosaic structure observed at the TPAMA_0326 and TPAMA_0488 loci would require multiple successive gene conversion events in both loci, which is unlikely. One possible explanation would presume a partial mosaic structure (Fig. 4) in both loci in the TPE donor DNA prior to crossing-over. Assuming this, the observed mosaic sequence at the TPAMA_0326 and TPAMA_0488 loci could result from a single gene conversion/recombination event.

Alternatively, there is a possibility of active DNA uptake across the cell membrane, which is more efficient, compared to natural competence of bacteria. Although no gene orthologs involved in natural competence have been identified in the TPA genomes, one cannot exclude this activity in one or more genes with unknown function. Internalization of TPE ssDNA would follow the model of mismatch repair.

TPAMA_0326 and TPAMA_0488 are mosaics resulting from interchromosomal recombination/gene conversion between TPA and TPE strains, while *tprC* and *tprD* alleles are the results of intrachromosomal recombination in *tprC* and *tprD* loci [12]. Therefore, similarities to TPE strains seen in *tprC* locus and TPAMA_0326 and TPAMA0488 loci arose via different mechanisms. Except for the TPAMA_0326 and TPAMA_0488 loci, two additional nucleotide positions (2 out of 1,192 single nucleotide changes differentiating TPA and TPE strains [7]; i.e. 0.168%) were found in the TP0314 locus and TPAMA_0319 gene. In these cases the Mexico A sequence was identical to the TPE sequences. These two nucleotide differences appear to represent differences that occurred by chance. For a single nucleotide position, the theoretical probability is 1,192/1,140,038*1/3 (i.e. 0.035%), where 1/3 is the probability that a particular nucleotide would be changed into a TPE nucleotide. Moreover, since the set of 1,192 single nucleotide changes that differentiate TPA and TPE strains is only based on comparisons of three TPA and three TPE strains, it is likely that the number of nucleotide positions differentiating all TPA and TPE strains will decrease with the newly reported whole genome sequences from other TPA and TPE strains.

Horizontal gene transfer (HGT) is an important process in bacterial evolution and the most frequently transferred genes usually bring selective advantage to the host cell. The TPA genome contains no prophages or IS-elements [28] or plasmids [29]. Nevertheless, the absences of modification and restriction systems together with the presence of genes for homologous recombination in TPA strains [4] appear to allow incorporation of foreign DNA molecules with subsequent integration into the chromosomal DNA. DNA transformation is commonly used in cultivable *Treponema denticola* [30] and related *Borrelia burgdorferi* strains [31]. Moreover, natural gene transfer among *Borrelia burgdorferi* has been observed [32]. In fact, 77 (8.32%) TPA genes were identified to be horizontally transferred by analysis of G+C contents, codon and amino acid usage, and gene position [17]. In our analysis, we did not find DNA regions of different G+C content to be associated with regions that differentiate TPA and TPE strains [7], nor were such associations found in *tpr* regions, indicating that the genome rearrangements took place before the diversification of these strains. It is therefore likely that the diversification of TPA and TPE strains was due to an accumulation of more subtle changes.

As shown by Centurion-Lara *et al.* [11], recombination mechanisms are more active during treponemal infection and gene conversion events represent important mechanisms for avoiding the host immune response. Therefore, uptake of TPE DNA by TPA strain, during a simultaneous TPA and TPE infection of a single host, with subsequent integration into TPA chromosome, appears to be a plausible explanation. Simultaneous infection with TPA and TPE is certainly possible during the early stages of syphilis infection. It has been shown that experimental infection with either TPA or TPE strains did not result in complete cross-protection, which suggests differences in the pathogenesis of syphilis and yaws [33], [34]. Although syphilis is preferentially transmitted sexually among adults, and yaws is preferentially transmitted via direct skin contact among children, simultaneous infection in a single host cannot be ruled out. The Haiti B strain, originally classified as a TPE strain due to having been isolated from “typical yaws lesions” in an 11-year-old child [13], has been recently reclassified as a TPA strain [19], [35], [36]. Moreover, Mexico A strain was isolated in a geographic region where both TPA and TPE infections occurred [37]–[39]. Nevertheless, recombination could also take place outside Mexico.

The mosaic TPAMA_0326 protein (Tp92) belongs to a relatively small group of treponemal outer membrane proteins [40] and is an ortholog of the BamA protein involved in outer membrane biogenesis [41]. BamA protein was identified as a TPA antigen exhibiting reactivity with sera from patients with syphilis [42], [43], and antibodies against this protein have opsonized living treponemes [44]. The 15 bp (TPE-like) deletion in the TPAMA_0326 influences the polyserine tract in a predicted large extracellular loop of TPAMA_0326 protein, which serves as a potential site for attachment to the host cells [44].

TPAMA_0488 encodes the methyl-accepting chemotaxis protein (Mcp2-1) [45]. Mcp2-1 is strongly expressed during experimental rabbit infections [46] and elicits a humoral response [45]. In the Mcp2-1 protein, there are 18 TPE-like changes, 8 of which are localized in the Cache domain [47], which binds small molecules during chemotaxis. All of these TPE-like changes cause amino acid changes, 7 non-conservative and 1 conservative. Taken together, due to described changes in extracellular/sensoring protein domains, both proteins can exhibit different antigenic epitopes and/or ligand binding activities.

Both TPAMA_0326 and TPAMA_0488 genes are under positive selection within TPA strains, as well as between TPA and TPE strains (genes were tested using codon-based testing by Čejková *et al.* [7]). The recombinant TPA strain (Mexico A) can thus possess a selective advantage in an infected host and could provide evasion from the host's immune system. However, it was recently shown that β-barrel structures, including surface-exposed loops of TPAMA_0326, where the TPE-like deletion is present, do not induce antibody response in humans [41], [48] On the other hand, positive selection need not be driven solely by the production of antibodies and may also comprise T-cell mediated cellular response, similar to the case of TprK [49]. In addition, positive selection operating on the periplasmic Cache domain of TPAMA_0488, recognizing small molecules, could reflect changed tissue tropism of TPE bacteria in comparison to TPA.

Despite selective advantage in the infected host (evasion from immune response, changed tissue tropism), these changes could result in the observed lower growth ability of the Mexico A strain compared to the Nichols strain under *in vitro* conditions [14]. Under positive selection, such a change can still have a growth advantage relative to the selective pressure on the host's immune system.

In summary, the mosaic character of the TPA Mexico A genome is likely the result of interstrain recombination between TPA and TPE strains during simultaneous infection in one host and similar patterns can be observed among other TP strains. These findings suggest the importance of horizontal gene transfer in the evolution of pathogenic treponemes.

## Supporting Information

Table S1 **List of genes encoding putative virulence factors annotated in the Mexico A genome.** Putative virulence factors were defined as those previously described by Čejková *et al.* [7] and comprised 31 genes (including *tpr*, *arp*, and TPAMA0136 genes).
(XLS)

Table S2 **Gene fusions identified in the Mexico A genome.** Frameshift mutations identified in the Mexico A genome resulted in gene fusions of 43 ORFs originally annotated in the SS14 genome. The resulting 21 ORFs annotated in the Mexico A genome are listed in this table. In stands for insertion and del for deletion.
(XLS)

Table S3 **ORFs containing sequence changes in comparison of the Mexico A genome with the Nichols genome.** Mexico A genome was compared with the Nichols genome (AE000520.1) and with the improved version of the Nichols genome (Pospíšilová, unpublished results). No change means that ORF sequence in the improved Nichols version was identical to the ORF in the Mexico A genome and the originally identified change was considered to be Nichols sequencing error. In the case of 14 Nichols ORF sequences, only partial (partial data) or no (N/A) sequencing data were available. Sub stands for substitution, in for insertion, del for deletion and MCS stands for multiple sequence changes.
(XLS)

Table S4 **Number of ORFs containing sequence changes in comparison of Mexico A with Nichols and SS14.** The Mexico A genome was compared with the Nichols genome (AE000520.1) and with the improved version of the Nichols genome (Pospíšilová, unpublished results). Numbers in brackets represent percentage of ORFs with sequence changes in each functional category.
(XLS)
